# Ultrafast olivine-ringwoodite transformation during shock compression

**DOI:** 10.1038/s41467-021-24633-4

**Published:** 2021-07-14

**Authors:** Takuo Okuchi, Yusuke Seto, Naotaka Tomioka, Takeshi Matsuoka, Bruno Albertazzi, Nicholas J. Hartley, Yuichi Inubushi, Kento Katagiri, Ryosuke Kodama, Tatiana A. Pikuz, Narangoo Purevjav, Kohei Miyanishi, Tomoko Sato, Toshimori Sekine, Keiichi Sueda, Kazuo A. Tanaka, Yoshinori Tange, Tadashi Togashi, Yuhei Umeda, Toshinori Yabuuchi, Makina Yabashi, Norimasa Ozaki

**Affiliations:** 1 Institute for Integrated Radiation and Nuclear Science, Kyoto University, Kumatori, Osaka Japan; 2 Institute for Planetary Materials, Okayama University, Misasa, Tottori Japan; 3 Graduate School of Engineering, Osaka University, Suita, Osaka Japan; 4 Graduate School of Science, Kobe University, Kobe, Hyogo Japan; 5 Kochi Institute for Core Sample Research, Japan Agency for Marine-Earth Science and Technology (JAMSTEC), Nankoku, Kochi Japan; 6 Institute for Open and Transdisciplinary Research Initiatives, Osaka University, Suita, Osaka Japan; 7 LULI, CNRS, CEA, École Polytechnique, UPMC, Univ Paris 06: Sorbonne Universités, Institut Polytechnique de Paris, Palaiseau, France; 8 SLAC National Accelerator Laboratory, Menlo Park, CA USA; 9 Japan Synchrotron Radiation Research Institute, Sayo, Hyogo Japan; 10 RIKEN SPring-8 Center, Sayo, Hyogo Japan; 11 Institute of Laser Engineering, Osaka University, Suita, Osaka Japan; 12 Joint Institute for High Temperatures RAS, Moscow, Russia; 13 Graduate School of Science, Hiroshima University, Higashihiroshima, Hiroshima Japan; 14 Center for High Pressure Science & Technology Advanced Research, Shanghai, China; 15 Extreme Light Infrastructure-Nuclear Physics, Magurele-Bucharest, ILFOV Romania

**Keywords:** Mineralogy, Mineralogy, Asteroids, comets and Kuiper belt

## Abstract

Meteorites from interplanetary space often include high-pressure polymorphs of their constituent minerals, which provide records of past hypervelocity collisions. These collisions were expected to occur between kilometre-sized asteroids, generating transient high-pressure states lasting for several seconds to facilitate mineral transformations across the relevant phase boundaries. However, their mechanisms in such a short timescale were never experimentally evaluated and remained speculative. Here, we show a nanosecond transformation mechanism yielding ringwoodite, which is the most typical high-pressure mineral in meteorites. An olivine crystal was shock-compressed by a focused high-power laser pulse, and the transformation was time-resolved by femtosecond diffractometry using an X-ray free electron laser. Our results show the formation of ringwoodite through a faster, diffusionless process, suggesting that ringwoodite can form from collisions between much smaller bodies, such as metre to submetre-sized asteroids, at common relative velocities. Even nominally unshocked meteorites could therefore contain signatures of high-pressure states from past collisions.

## Abstract (web summary)

Meteorites from space often include denser polymorphs of their minerals, providing records of past hypervelocity collisions. An olivine mineral crystal was shock-compressed by a high-power laser, and its transformation into denser ringwoodite was time-resolved using an X-ray free electron laser.

## Introduction

Primordial solid materials in the solar system, such as interplanetary dust collected in the Earth’s stratosphere, asteroid regolith recovered by the Hayabusa spacecraft, and chondritic meteorites that have fallen to the Earth, commonly have olivine [α−(Mg,Fe)_2_SiO_4_] as their major component mineral^1–4^. These primordial materials are the building blocks of planetesimals that accreted to form terrestrial planets, leaving olivine as one of the main mineral components of the Earth. The high-pressure polymorphs of olivine are thus expected to have played a major role in the deep Earth^5^. This idea was confirmed by static high pressure experiments and geological studies^6,7^, which demonstrated that 410–660 km below the Earth’s surface, where material is subject to pressures from 14 to 23 GPa, the mantle mostly consists of wadsleyite [β−(Mg,Fe)_2_SiO_4_] and ringwoodite [γ−(Mg,Fe)_2_SiO_4_]. Meanwhile, planetary scientists have discovered these same polymorphs in shocked chondritic, lunar and Martian meteorites, suggesting that pressures comparable to those in the Earth’s deep interior were generated in space environments by impact events^8,9^.

The first example of such high-pressure polymorphs in extra-terrestrial materials were granular crystals of ringwoodite found in a chondritic meteorite named Tenham^10^. Assuming a chemical diffusion model within the crystals, their growth was estimated to have continued for approximately one second at high pressure^11^. Later, sub-micrometre-thick planar crystals of ringwoodite were repeatedly discovered from shocked olivine in other chondritic meteorites, which were also estimated to require duration of several seconds at high pressure for the transformation to proceed^12–14^.

Most recently, a third high-pressure polymorph of olivine was discovered in the Tenham meteorite^15^, and has been recognised as a new mineral ‘poirierite’ [ε−(Mg,Fe)_2_SiO_4_] by the International Mineralogical Association^16^. Poirierite was previously predicted to recrystallise as a transient structure when olivine transforms into wadsleyite or ringwoodite in the solid state^17^. However, the poirierite had grown as numerous parallel plates with thicknesses on the order of nanometres, within the already-discovered granular crystals of ringwoodite. It apparently recrystallised just after supercooling, either at peak shock pressure or during the subsequent pressure release after the impact event^15,16^. This could result in different information about the impact processes being retained, compared to what can be learnt from the host ringwoodite crystals with granular morphologies.

From the analyses of recovered natural samples, and on the basis of static high pressure experimental results, it had been proposed that the high pressure polymorphs were formed over several seconds, at shock pressures up to ~25 GPa (e.g. 14–25 GPa for ringwoodite formation, corresponding to the impact velocity around 2 km/s^12,18^). Alternatively, the time and pressure scales may be determined by conducting dynamic compression experiments of planetary minerals in the laboratory. In a previous experiment, post-compression sample analysis suggested a reaction duration of sub-milliseconds for crystal growth of wadsleyite^19^, but was not able to observe the crystal growth during the compression. Recently, using strong laser pulses to induce dynamic compression, thermodynamic properties of compressed planetary minerals^20–22^ as well as their crystallisation kinetics^23^ have been directly determined via in-situ analysis.

In this work, we use such strong laser pulses in combination with ultrashort x-ray pulses. We find a novel, ultrafast transformation mechanism of olivine within the shock-induced high-pressure state. An ultrafast and coherent shearing of oxygen layers are confirmed to occur, the first time such a process is observed within an oxide crystal, with significant implications for shock environments in the past.

## Results

### A denser structure emerging within nanoseconds

**Fig. 1:**
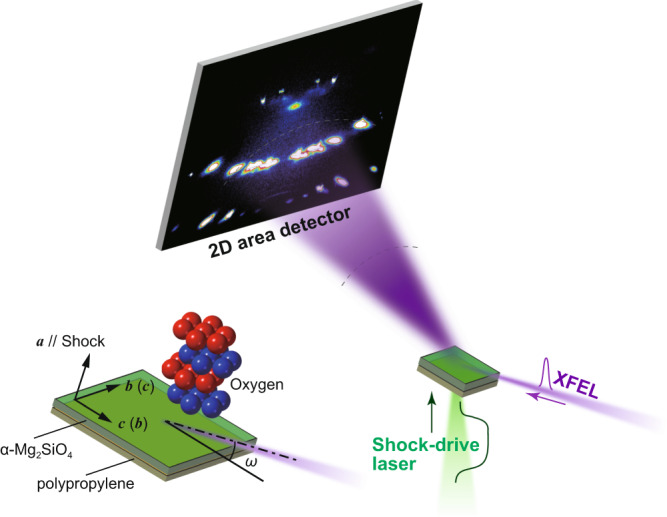
Experimental system. The α − Mg_2_SiO_4_ single crystal was shock-compressed along its crystallographic *a*-axis by irradiation of the power laser pulse into an ablator of polypropylene film, where the crystal structure was simultaneously analysed using the XFEL pulse. A thin polycrystalline Al_2_O_3_ plate (not shown) was fixed on the upper surface of α − Mg_2_SiO_4_ when the shock arrival time was evaluated.

**Fig. 2:**
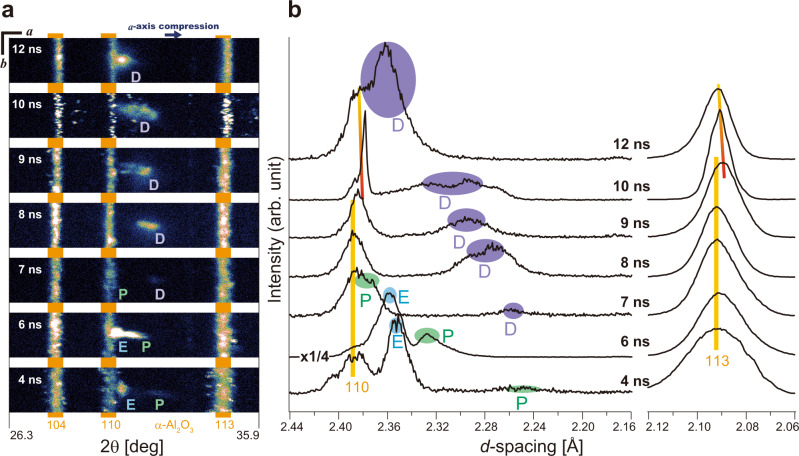
XFEL diffraction results showing time evolution of *g* = 200 reflections from the polypropylene-olivine-Al_2_O_3_ layered target. **a** Two dimensional images where each number represents the delay time *t* of an XFEL pulse from the power laser pulse arrival time to the surface of polypropylene. Diffuse spots marked ‘E’, ‘P’, and ‘D’ show the reflection from the oxygen layers of olivine in elastic and plastic shock wave regions, and reflection from its denser recrystallised region, respectively. Crystallographic orientations (*a* and *b*) are approximately shown for readability, together with the direction of *a*-axis compression. These axes are not necessarily precisely located on the detector plane. A vertical linear profile within the three orange strips with the Laue indices shows Bragg reflections from the (104), (110), and (113) planes of polycrystalline Al_2_O_3_. **b** One dimensional patterns as a function of *d* obtained by the integration of the images. Positions of the peaks marked with E, P and D show *d* of their corresponding reflections, *d*_200_^E^, *d*_200_^P^, and *d*_200_^D^, respectively. At *t* = 4 ns, the *d*_200_^P^ was compressed to 94 ± 1 % of *d*_200_^1atm^, which was comparable to the *a*-axis compression at static pressure of 60 to 100 GPa^57,58^. At *t* = 6 ns, the E and P peaks become much stronger due to the increase in volume of these compressed regions (the shown intensity profile was reduced by a factor of 4). At *t* = 7 ns, the ‘E’ and ‘P’ peaks moved further to the left to become almost undetectable (see ‘Methods’), while the ‘D’ peak started to grow (*d*_200_^D^ = 2.25−2.26 Å, corresponding to *d*_222_ of ringwoodite at 21 to 25 GPa). At *t* = 8 ns, the newly-emerged ‘D’ peak rapidly grew; it was also gradually shifting to the right (*d*_200_^D^ = 2.25 to 2.31 Å, corresponding to *d*_222_ of ringwoodite at 6 to 25 GPa). At *t* = 9 ns, the reflections of Al_2_O_3_ suddenly became slightly compressed, indicating that the shock wave had travelled throughout the olivine plate to arrive there. The strips with orange (not compressed) and red (compressed) colours are visual guides for the behaviour of these Al_2_O_3_ peaks.

Figure 1 schematically represents the experimental system for laser-driven shock compression, which is installed and operated at the SACLA (SPring-8 Angstrom Compact Free Electron Laser) facility. We first determined the speed of olivine’s transformation into its polymorph. Fig. 2a shows the time evolution of a Bragg reflection spot (*g* = 200), which came from the crystal structure of olivine, as observed in the series of XFEL diffraction images detected by a high-space resolution multiport charge-coupled device (MPCCD, see Methods). As the Bragg equation 2*d*sin*θ* = *λ* indicates, the diffraction angle 2*θ* probes the separation of adjacent oxygen layers, *d*_200_. Upon propagation of the shock wave, *d*_200_ was compressed from its original value, *d*_200_^1atm^ = 2.378 Å. The *g* = 200 reflection then consistently shifted towards the ‘*a*-axis compression’ direction, as seen in these two-dimensional detector images. The integrated one-dimensional diffraction patterns as a function of *d* = *λ*/2sin*θ* show the time evolution of *d*_200_ in a quantitative manner (Fig. 2b). The laser pulse begins at *t* = 0, and after 4 ns the shock had passed thorough the polypropylene ablator to arrive at the olivine sample crystal. Two diffuse reflections, marked ‘E’ and ‘P’, simultaneously emerged at the positions *d*_200_^1atm^ > *d*_200_^E^ > *d*_200_^P^. These reflections came from elastic (E) and plastic (P) shock waves travelling through the olivine. As the shock wave decays, *d*_200_^E^ and *d*_200_^P^ expanded and then disappeared when they lost the reflection condition (see Methods). At *t* ≥ 7 ns, another diffuse reflection, marked ‘D’, emerged and grew at the position *d*_200_^P^ > *d*_200_^D^. This distinctly smaller *d*_200_^D^ —compared to those from olivine in the elastic and plastic waves—must be due to denser atomic packing of the new crystal structure having an Mg_2_SiO_4_ composition; it is most likely one of wadsleyite (β), ringwoodite (γ), or poirierite (ε). The coexistence of mechanically distinct regimes within a shock-compressed Mg_2_SiO_4_ single crystal was previously reported by means of a conventional shock-velocity measurement scheme^24,25^, where three independent shock velocities were determined and ascribed to be those of elastic (olivine), plastic (olivine), and plastic (denser structure) waves^25^. We thus generated and analysed the expected shock wave structure with sufficient time resolution for resolving the in-situ transformation of olivine.

We note that the ‘D’ reflection, corresponding to the denser polymorph, significantly grew within a few nanoseconds, between *t* = 6 ns and *t* = 8 ns. Although such fast growth of a dense SiO_2_ polymorph from its glass state was previously observed within 1.4 ns by a laser-driven shock experiment^23^, the time window of <2 ns is too short for nucleation and growth of any dense polymorph structures of Mg_2_SiO_4_ because it involves two different cation species (Mg^2+^ and Si^4+^); to activate the conventional nucleation and growth mechanism of such a complex oxide, it is necessary to rearrange both cations and the anion (O^2-^), which has never been observed on such a short timescale. Moreover, as seen in Fig. 2a, the original direction of the oxygen layers was kept in the transformed structure, which would not agree with a transformation occurring by nucleation and growth. Another possible rearrangement mechanism from the olivine (α) structure of Mg_2_SiO_4_ into the denser polymorphs was theoretically proposed^17^, which works without any thermal diffusion of atoms (i.e. it is a diffusionless process), and which directly yields either a ringwoodite (γ) or poirierite (ε) structure. This mechanism proposes that shearing of the whole crystal lattice may play an essential role when olivine is compressed along the ‘*a*-axis compression’ direction^17^ as in the current experiment.

### Ringwoodite recrystallisation through ultrafast lattice sharing

**Fig. 3:**
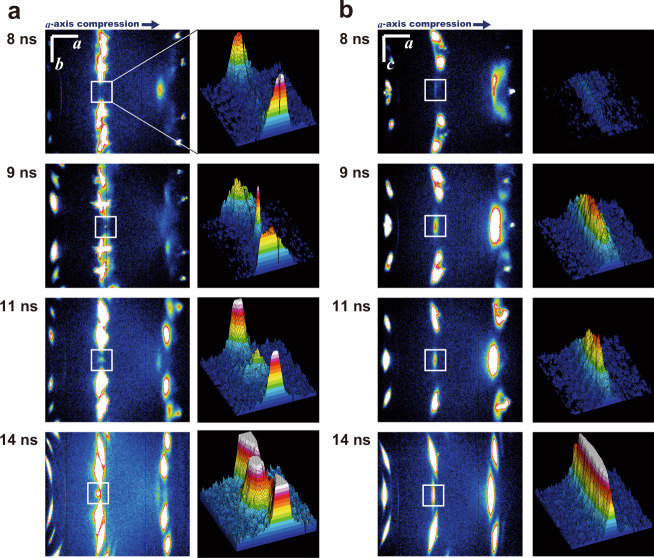
XFEL diffraction images showing time evolution of *g* = 300 reflection from the polypropylene-olivine layered target. Crystallographic orientations (*a*, *b*, and *c*) are approximately shown for readability together with the direction for *a*-axis compression. These axes are not necessarily exactly located on the detector plane. White squares show the position of *g* = 300, and their magnified views were separately shown at the right of the images. **a** Series images along the *b* direction, indicating that the *g* = 300 reflection was not deformed. **b** Series images along the *c* direction, indicating that the *g* = 300 reflection was extensively deformed. Laue indices of the other reflections together with the images of wider area coverage are shown in Supplementary Fig. 3.

**Fig. 4:**
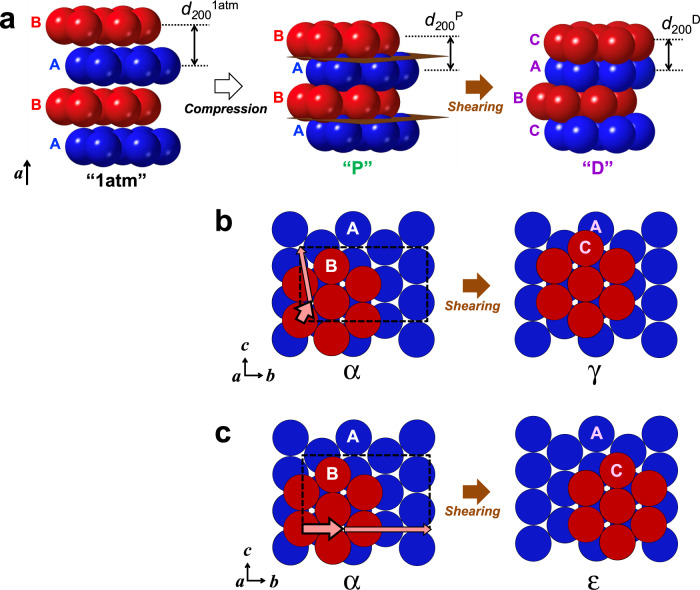
Schematic illustrations of shearing mechanism of Mg_2_SiO_4_. **a** Perspective views of oxygen anion layers, showing how the original α structure (1 atm) was compressed along its *a*-axis (P), and then recrystallised into the dense structure (D) induced by fast shearing (slipping) motions of these layers at the slip planes shown with brown colour. The original α structure consists of alternating A and B layers of oxygen forming a hexagonal close packing (hcp) arrangement. The dense polymorph structure (β, γ, or ε) all consists of alternating A, B, and C layers of oxygen forming a cubic close packing (ccp) arrangement. **b** The shearing model toward the γ structure, where cooperative slip motions of the oxygen layers occur along the *c* axis of the α structure, [001]_α_. Only the oxygen anions are shown for clarity. See Supplementary Fig. 4 for the accompanying cooperative motions of magnesium and silicon cations. The unit cell of α structure is indicated by the rectangle with dotted lines. The upper layer B slips with respect to the lower layer A by partial dislocation with Burgers vector **b**_**p**_ = 1/12[013]_α_, as indicated by the thick solid pink arrow. The other partial dislocations were indicated by the thin solid pink arrow, where all these partial dislocations in total constitute the perfect dislocation with the Burgers vector **b**_**t**_ = [001]_α_ that defines the direction of macroscopic shearing along the *c* axis^17,26^. **c** The shearing model toward the ε structure, where cooperative slip motions of the oxygen layers occur along the *b* axis of the α structure, [010]_α_. The unit cell of α structure is shown by the rectangle with dotted lines. The layer B slips with respect to the layer A by partial dislocation with **b**_**p**_ = 1/3[010]_α_, as indicated by the thick solid pink arrow. The other partial dislocations were indicated by the thin solid pink arrow, where all these partial dislocations in total constitute the perfect dislocation with **b**_**t**_ = [010]_α_ that defines the direction of macroscopic shearing along the *b* axis^17^.

To determine the most plausible polymorph candidate for the recrystallisation from among β, γ, and ε phases, Bragg reflections other than *g* = 200 of the original α phase were observed and analysed by a separate series of experiments using a flat panel X-ray detector (FPD, see ‘Methods’). Fig. 3 shows the evolution of representative two-dimensional detector images around the *g* = 300 position of α, which proved to be particularly informative for determining the resulting polymorph. This reflection originally showed zero intensity, owing to the crystallographic reflection conditions for the structure of α (see Methods). This was fully consistent with the results observed before at *t* = 8 ns. After this point, however, the intensity of *g* = 300 significantly increased with time. Because the intensity of the ‘D’ reflection in Fig. 2b also increased after *t* = 8 ns, we infer that the crystal structure responsible for ‘D’ must have an appropriate space group and unit cell parameters to induce this reflection. Both the β and γ structures satisfy these criteria, while the ε structure does not (see ‘Methods’). In addition, as established by the theoretical study on conceivable shear transition mechanisms of Mg_2_SiO_4_^17^, the β structure may recrystallise only after the ε structure crystallises through shearing of the α structure, whereas the γ may recrystallise directly. This is because the β structure does not closely conform to the α structure in terms of its cation arrangements, while the γ structure is very close^15,17,26^. Thus, the existence of the *g* = 300 reflection indicates that the product was either β via ε, or γ. To further discriminate between these two possibilities, we note that the reflection did not have a circular profile, as is expected in case for a shear-free lattice^27^, but was instead distorted along the direction corresponding to that of the *c*-axis of the α structure, while not being diffused along that of the *b*-axis (Fig. 3). The diffuse geometry of reflection indicates non-uniform interlayer distances between the crystallographic planes along the elongated direction. If the γ structure is recrystallised from α, then the oxygen layers slide along the *c*-axis, whereas if the β structure is recrystallised through ε as the intermediate from α, then the oxygen layers slide along the *b*-axis first (Fig. 4). The observed shearing direction is along the *c*-axis, and we therefore concluded that the polymorph formed was γ−Mg_2_SiO_4_. As described, this is the most frequently observed high-pressure mineral phase occurring in the shocked meteorites, and that confirms its possible origin in transient compression events occurred in the solid state.

## Discussion

The possible origin of ringwoodite (γ) as the consequence of a fast lattice-shearing mechanism has important implications in the shock history that occurred in the solar system. In general, high-pressure mineral polymorph structures can be formed by one of two rearrangement mechanisms: either nucleation and growth (reconstructive, with atomic diffusion), or lattice-shearing (pseudomartensitic, without atomic diffusion)^28–30^.

In the former mechanism, the product crystals nucleate at grain boundaries or defects in the original structure. Thus, the products tend to have random crystallographic orientations which are not constrained by the original lattice direction^28,30^. This mechanism works for structural transformations in every type of material, but only becomes significant at temperatures close to the melting point, and occurs on timescales of a few seconds, as seen when granular crystals of ringwoodite were growing at the grain boundaries^31,32^ or even in the melt^32,33^.

In the latter mechanism, the product crystals nucleate inside the original crystals, away from grain boundaries or defects. In the case of oxides and silicates, this mechanism involves lattice-coherent fast shearing process of the oxygen sublattice, with only slight cation displacements^28–30^. Such shearing transitions were first identified to work in steel (Fe-C). Where excess stresses exist, the mechanism may proceed quite effectively even at much lower temperatures than the melting points of the system, and is proposed to trigger the recrystallisation of planar or lens-shaped crystals of ringwoodite inside the host olivine grains^28–30^. In addition, lattice shearing tends to orient the newly-formed crystals towards characteristic crystallographic directions, namely, topotaxy. Thus the transformation results in a coherent geometrical relationship between the original and the product crystal lattices, as seen in our current results (Fig. 2a).

While both granular and planar type crystal morphologies were observed in the shocked meteorites, the current results indicate that the planar type could recrystallise on much shorter timescales than any previous prediction or estimation^11–14,19,34,35^. This ultrafast mechanism could form high-pressure polymorphs even if pressure and stress increased and decreased within milliseconds or shorter timescales, as would be expected for impacts between metre to submetre-sized bodies^8,19^. In fact, because the recrystallisation was triggered and proceeded during the release stage of the shock events (Fig. 2), there is potentially no minimum requirement on the shock duration, as long as the initial pressure reaches the threshold.

Such an ultrafast mechanism, and the methodology for how to time-resolve it, may provide novel implications for the future research direction of developing functional engineering ceramics. For example, the lattice-shearing transformation plays for a significant role in ‘transformation toughening’ of ZrO_2_-based ceramic alloys^36,37^, where a faster mechanism minimises microcracking and thus increases the toughness. Another example is in the transformation of Ga_2_O_3_^38^, which involves collective shearing of close-packed oxygen layers, and short-range migration of Ga cations across these layers; these processes are identical to the case for Mg_2_SiO_4_ observed in the current experiments. Analysis of recovered Ga_2_O_3_ at ambient pressure after shock compression showed that the transformation could proceed within hundreds of nanoseconds^39^. These examples in ceramic systems support the emerging idea to expect that ultrafast transformations under stress may play a wide variety of roles in designing functional oxide materials.

Finally, we consider the possible role of this ultrafast mechanism in inferring the shock history of the early solar system. The crystallisation of ringwoodite in shocked meteorites was believed to occur at pressures around its equilibrium phase boundary (14–25 GPa)^12–14,40^, as determined by static experiments at high pressure and temperature conditions. The impact velocities were estimated to be ~2 km/s^12,18^, which would be common among planetary embryos towards the late stages of planetary formation^41^. Because of the larger sizes, the high pressure would be maintained for several seconds, which was sufficient to trigger the conventional nucleation and growth mechanism of ringwoodite during the relevant impact events.

On the other hand, planetary scientists have suggested that meteorites with evidence of the most intense impact events in the solid state have experienced peak pressures up to 60−75 GPa^42,43^. From the lattice compression of α−Mg_2_SiO_4_, we estimate that the peak pressures reached in the current experiment were 60−100 GPa (Fig. 2b). In both cases, the pressures are considerably higher than the equilibrium phase boundary of 14–25 GPa for ringwoodite crystallisation. We note that the ultrafast structure transformation did not occur at this peak pressure, but instead while the shock was releasing, after the pressure had decreased close to or even below the thermodynamic equilibrium phase boundary. This is possibly because the relevant slip planes were kept immobile at the peak pressure due to the high normal stresses loaded to these planes, and then became mobile as the stress decreased with shock propagation. If ultrafast structure transformation mechanisms are in general triggered during the shock release, then previously-inferred peak pressures from conventional, solid-state crystal growth kinetics of ringwoodite^12,14,35^ would have been significantly underestimated.

It is important to note that, due to the short timescale of the described transformation mechanism, it can be triggered by even metre to submetre-sized asteroid impacts if their relative velocity satisfies the criteria. The Hugoniot data of an ordinary chondrite and a basalt indicates that shock pressures of 60 and 100 GPa, the most probable pressures to trigger the olivine to ringwoodite transformation, are produced by their impacts with relative velocities of 5 and 7 km/s, respectively^8,44^, some of the most common values observed in the current asteroid belts^45^. We would therefore expect records of ringwoodite formation to be much more prevailing than previously expected, and that exploration of even nominally unshocked meteorites might find sub-micrometre-thick lamellar ringwoodite.

Two microstructural types of collision records in meteorites may therefore be discriminatively analysed; asteroid collisions would lead to microstructures formed by the ultrafast mechanism, while those of larger planetary embryos would form structures by the conventional mechanism. Although the former have not been intensively explored in previous meteorite research, we believe that such signatures may be present even in those meteorites that have been nominally categorised as unshocked. The regolith materials of present asteroids would have also recorded their recent impact events with relative velocities between 5 and 7 km/s. The JAXA Hayabusa mission concluded that the subkilometre-sized asteroid Itokawa, for instance, has experienced multiple large impact events on the basis of morphological analysis of its surface^46^. The mission successfully took samples from the surface of Itokawa^3,4^, which were proved to consist of ordinary chondritic materials. We expect these samples to have recorded signatures of the ultrafast mechanism, because the shock pressure duration on subkilometre-sized asteroid is not sufficient to trigger the conventional mechanism. Further nano-scale investigations of nominally unshocked meteorites and recovered asteroid samples in the past and in the future will shed light on the hidden high-pressure events occurring throughout the evolution history in the solar system.

## Methods

### Experimental Setup

The experiment was conducted in the experimental hatch EH5 at the beamline BL3 of SACLA^47^ (Fig. 1). A custom-made power laser system (Hamamatsu Photonics K. K., Japan) generated visible laser pulses of 532 nm in wavelength. The shot-to-shot laser energy and pulse shape were monitored throughout the experimental campaigns. The energies were 4.2 ± 0.2 J (~5%) for Fig. 2 series and 7.5 ± 0.5 J (~7%) for Fig. 3 series. The pulse shape was a quasi-square with 3.3 ± 0.2 ns full width at half maximum (FWHM) and with repeatable rise time (1 ± 0.1 ns). The 4.2 J and 7.5 J energy pulses were focused into a ~250 μm or slightly smaller diameter spot to have ~2 × 10^12^ W/cm^2^ laser power density on the target. This pulse ablated a 30 ± 1 μm thick polypropylene film, driving a shock through the film and into a 50 ± 1 μm thick single-crystal plate of forsterite [α−Mg_2_SiO_4_] olivine. On some shots, an additional 15 μm thick polycrystalline Al_2_O_3_ was glued to the rear side of the α−Mg_2_SiO_4_ crystal for sensing the shock arrival after it passed through α−Mg_2_SiO_4_ (See Supplementary Fig. 1). In this case, the 200 diffraction spot of the shock-compressed α−Mg_2_SiO_4_ single crystal can be easily discriminated from the Debye ring of (110) of Al_2_O_3_, even though they originally have a similar 2*θ* (Fig. 2). When the shock arrived at Al_2_O_3_ after propagating through 50 μm thick of α−Mg_2_SiO_4_, the pressure had already decayed down to a few GPa (Fig. 1b after *t* = 9 ns), possibly because the following rarefaction wave caught up the main wave, which was suggested by the results of one-dimensional hydrodynamic simulations.

The shock wave evolution and pressures reached were estimated by one-dimensional radiation hydrodynamics simulations conducted using the MULTI code^48^ at our applied laser conditions (see Supplementary Fig. 2). The simulations reproduced the basic time-evolution features of the shock, such as its arrival times at the ablator/α−Mg_2_SiO_4_ interface at 3–4 ns and at the α−Mg_2_SiO_4_ back surface at 8 to 9 ns. The simulated peak pressure of the sample was ~100 GPa and consistent with the observed lattice compression (60–100 GPa, Fig. 2) when the shock front arrived at the sample. These reproduced features by the simulations indicated that the experimentally-observed shock profile was well approximated as one-dimensional. Otherwise, the resulting profile would be more heterogeneous and not consistent with the simulations. This interpretation was also supported by time-resolved shock emission signals observed by a streaked optical pyrometer, indicating that a good planar shock of approximately 200 μm in diameter was generated at the ablator/α−Mg_2_SiO_4_ interface, where the sample was analysed by X-ray free electron laser (XFEL).

The rearrangement processes of crystal structures during the shock wave propagation were probed using XFEL pulses generated at SACLA facility. Each XFEL pulse has a photon energy of 9.99 ± 0.01 keV (wavelength *λ* = 1.24 Å) with an energy bandwidth of *ΔE*/*E* ~ 0.5%, 0.2–0.4 mJ of pulse energy, and an FWHM pulse duration of ~10 fs^49^. Note that this energy bandwidth occasionally broadened due to technical reason at the XFEL source (as seen in Fig. 2a at *t* = 4 ns), as confirmed by monitoring of XFEL source profile. We synchronised the timing of the high-power laser pulse and the XFEL pulse with variable delays using the scheme already described elsewhere^50,51^. The root-mean-square timing jitter of ±16 ps in the high-power laser system, relative to the XFEL pulse timing, is anticipated by its specification, and is not large enough to affect the results here. The thickness of the glue between polypropylene, α−Mg_2_SiO_4_, and Al_2_O_3_ was kept <0.5 μm, which only induced <100 ps of arrival time delays to induce trivial effects on the timing of shock propagation. We used an X-ray camera and a centre pin in combination for finding the XFEL beam position, and then used a CCD camera and the same pin to overlap it with the shock-driving laser. The XFEL beam was vertically focused to ~15 μm width using a Kirkpatrick–Baez mirror system where much better pointing accuracy than 10 μm was achieved. The focused XFEL beam was pointed for this width of α−Mg_2_SiO_4_ volume located at the closest distance to the ablator. By using this vertically-focused beam geometry and by adopting a reflection geometry of XFEL, which was obliquely injected from the other side of the sample in reference to the shock-driving laser (Fig. 1), we selectively analysed the most-compressed volume of the sample crystal as a function of time (see Supplementary Fig. 1). The XFEL beam was horizontally sliced into ~200 μm width using a two-quadrant slit for Fig. 2 series, or it was horizontally focused into 20−30 μm width using another K-B mirror for Fig. 3 series. In the latter case, the horizontal pointing accuracy was 30 μm or better. The planar shock front size of ≥200 μm in diameter at the ablator/α−Mg_2_SiO_4_ interface was therefore set comparable or smaller than the horizontal XFEL beam sizes throughout all the experiments.

The grazing angle *ω* between the target surface and the XFEL beam was set to satisfy the diffraction condition of the desired reflection from the single crystal (Fig. 1). There exists a small sample-by-sample fluctuation of *ω*, typically within ±1.0°, which was mostly because of the uncertainty of crystal orientation hand-adjusted under optical microscopes. This is not trivial and we mechanically realigned each sample crystal to have the accurate *ω* against XFEL beam path prior to each shock experiment. As an example, in order to observe the behaviour of *g* = 200 reflection of α−Mg_2_SiO_4_ at *θ* = 15.10°, we maximised the reflection intensity by manually scanning the *ω* within 15.1 ± 0.1° window (typically with 0.05° resolution) with the support of continuous XFEL pulse supply. Once the intensity of reflection from the non-compressed crystal lattice was maximised, we arbitrary increased the *ω* for maximising the observed intensity from the compressed lattice, and conducted the shock experiment. This procedure was also effective in protecting the detector from destruction induced by a strong reflection from the non-compressed portion of the target crystal lattice. Since the non-compressed lattice did not satisfy the diffraction condition, it did not induce any signal to the detector images.

To complete the dataset acquisition at one specific delay time *t*, we step-by-step increased the *ω* (target tilt angle) until no intensity was detected even for the compressed lattice of the targeted reflection indices. Therefore, we have shot data with each *ω* and each driving laser energy at least. Thus, total number of the shot data was an order of magnitude larger than those shown in Figs. 2 and 3, which in total will be reported elsewhere in the future separately. The ablator/α−Mg_2_SiO_4_ interface was set focused by the XFEL beam, which therefore travelled through ~200 μm (= 50 μm/sin 15°) of distance from the backside of the crystal, in case we focus to *g* = 200 (Fig. 1). This travel distance is almost equal to the attenuation length of 10 keV X-ray through the medium. A dual-sensor MPCCD detector with 50 × 50 μm^2^ pixel size and 51.2 × 51.2 mm^2^ detector size^52^ was used for the analysis of the *g* = 200 reflection. It was set obliquely upward from the sample position with a camera length of 300.7 mm and 2*θ* coverage of 31 ± 5°. The images shown in Fig. 2a (180 × 1000 pixels) were cropped from the full MPCCD images (1024 × 1024 pixels), without losing the described coverage of 2*θ*. The variability of incident XFEL pulse energy induced an apparent intensity fluctuation of the detector images, which was normalised using the integrated intensity of (113) reflections of Al_2_O_3_ throughout the image (Fig. 2b). In Fig. 2, we showed the images obtained at the grazing angle *ω* arbitrary increased by +0.5° (*t* = 6 ns), + 1.0° (*t* = 4 ns, 7 ns, 8 ns and 12 ns), or +1.5° (*t* = 9 ns and 10 ns) from the exact 2*d*_200_^1atm^sin*ω* = λ condition. Due to this setup, the reflection from only slightly compressed olivine crystals (*d* ~ *d*_200_^1atm^) was not detectable. We did not observe any reflection from the ablator or its transformed products such as diamond. A wide-area flat-panel detector (FPD; Rad-icon) with a detector size of 204 × 153 mm^2^ and a camera length of 76.5 mm was used for the analyses of *g* = 300 reflections. The selected-area images of 54 × 63 mm^2^ dimensions taken with this detector are shown in Fig. 3, which covers 2*θ* from 28° to 70° and *d*-spacing from 2.55 to 1.08 Å (see Supplementary Fig. 3). The two detectors were separately calibrated for their optical geometries using diffraction patterns of standard materials (Au and/or Al_2_O_3_, see Supplementary Fig. 3) and the IPAnalyzer software^53^. Reciprocal geometries and reflection intensities in the detector images were forecasted and analysed using the ReciPro software^54^ where we successfully simulated the expected diffraction patterns coming from the prefixed geometry of XFEL beam direction, sample crystal lattice orientation, and detector geometry.

### Assignments of reflections in XFEL diffraction images

Diffraction spots from single-crystal olivine and its polymorphs under shock compression were indexed considering *d*-spacings and inter-angles of reciprocal lattice vectors of respective unit cells. The reflection conditions were evaluated based on the crystal symmetry using the following crystallographic parameters for a pure Mg_2_SiO_4_ composition, as follows: Olivine: space group, *Pbnm* (orthorhombic); lattice parameters, *a* = 4.756 Å, *b* = 10.195 Å, *c* = 5.981 Å (supplied from Oxide Corporation, Hokuto, Yamanashi, Japan); reflection conditions, *k* = 2*n* for 0*kl* (*b*-glide plane; *n* is an integer), *h* + *l* = 2*n* for *h*0*l* (n-glide plane). Wadsleyite (spinelloid): space group, *Imma* (orthorhombic); lattice parameters, *a* = 5.698 Å, *b* = 11.438 Å, *c* = 8.257 Å;^55^ reflection conditions, *h* + *k* + *l* = 2*n* for *hkl* (body-centred lattice), *h* = 2*n* for *hk*0 (*a*-glide plane). Ringwoodite (spinel): space group, *Fd-*3*m* (cubic); lattice parameters, *a* = 7.997 Å at 5.7 GPa, 7.831 Å at 21.0 GPa, and 7.785 Å at 25.1 GPa;^56^ reflection conditions: *h* + *k*, *k* + *l*, *h* + *l* = 2*n* for *hkl* (face-centred lattice), *h* + *k* = 4*n* for *hk*0, *k* + *l* = 4*n* for 0*kl*, *h* + *l* = 4*n* for *h*0*l* (*d*-glide plane). Poirierite (spinelloid): space group, *Pmma*; lattice parameters, *a* = 5.698 Å, *b* = 2.860 Å, *c* = 8.257 Å (assumed based on the lattice parameters of wadsleyite);^15^ reflection conditions, *h* = 2*n* for *hk*0 (*a*-glide plane).

## Supplementary information

Supplementary Information

## Data Availability

The raw data generated during the x-ray diffraction experiment are available from the corresponding author on reasonable request.

## Code availability

The software codes can be found at the referred website.
