# Supplementary material for: Ultrafast olivine-ringwoodite transformation during shock compression
Source: Nat Commun. 2021 Jul 14;12:4305. doi: 10.1038/s41467-021-24633-4 (PMC8280208; doi:10.1038/s41467-021-24633-4)
Supplement: Supplementary file 1 — Supplementary Information [file 41467_2021_24633_MOESM1_ESM.pdf]

Supplementary information for

Ultrafast olivine-ringwoodite transformation during shock compression

Takuo Okuchi[1,2,3]*, Yusuke Seto[4], Naotaka Tomioka[5], Takeshi Matsuoka[6], Bruno Albertazzi[3,7], Nicholas J. Hartley[3,8], Yuichi Inubushi[9,10], Kento Katagiri[3], Ryosuke Kodama[3,11], Tatiana A. Pikuz[3,6,12], Narangoo Purevjav[2], Kohei Miyanishi[10,11], Tomoko Sato[13], Toshimori Sekine[3,14], Keiichi Sueda[10], Kazuo A. Tanaka[3,15], Yoshinori Tange[9], Tadashi Togashi[9,10], Yuhei Umeda[1,2,3,11], Toshinori Yabuuchi[9,10], Makina Yabashi[9,10], and Norimasa Ozaki[3,11]

[1] Institute for Integrated Radiation and Nuclear Science, Kyoto University, Kumatori, Osaka 590-0494, Japan

[2] Institute for Planetary Materials, Okayama University, Misasa, Tottori 682-0193, Japan

[3] Graduate School of Engineering, Osaka University, Suita, Osaka 565-0871, Japan

[4] Graduate School of Science, Kobe University, Kobe, Hyogo 657-8501, Japan

[5] Kochi Institute for Core Sample Research, Japan Agency for Marine-Earth Science and Technology (JAMSTEC), Nankoku, Kochi 783-8502, Japan

[6] Institute for Open and Transdisciplinary Research Initiatives, Osaka University, Suita, Osaka 565-0871, Japan

[7] LULI - CNRS, CEA, Sorbonne Universités, Ecole Polytechnique, Institut Polytechnique de Paris - F-91128, France

[8] SLAC National Accelerator Laboratory, 2575 Sand Hill Road, Menlo Park, CA, 94025, USA

[9] Japan Synchrotron Radiation Research Institute, Sayo, Hyogo 679-5198, Japan

[10] RIKEN SPring-8 Center, Sayo, Hyogo 679-5148, Japan

[11] Institute of Laser Engineering, Osaka University, Suita, Osaka 565-0871, Japan

[12] Joint Institute for High Temperatures RAS, Moscow, 125412, Russia

[13] Graduate School of Science, Hiroshima University, Higashihiroshima 739-8526, Japan

[14] Center for High Pressure Science & Technology Advanced Research, Shanghai, 201203, China.

[15] Extreme Light Infrastructure-Nuclear Physics, Str. Reatorului 30, Magurele-Bucharest, ILFOV 077125, Romania

*Corresponding author: Phone: +81-72-451-2474; Email: okuchi@rri.kyoto-u.ac.jp

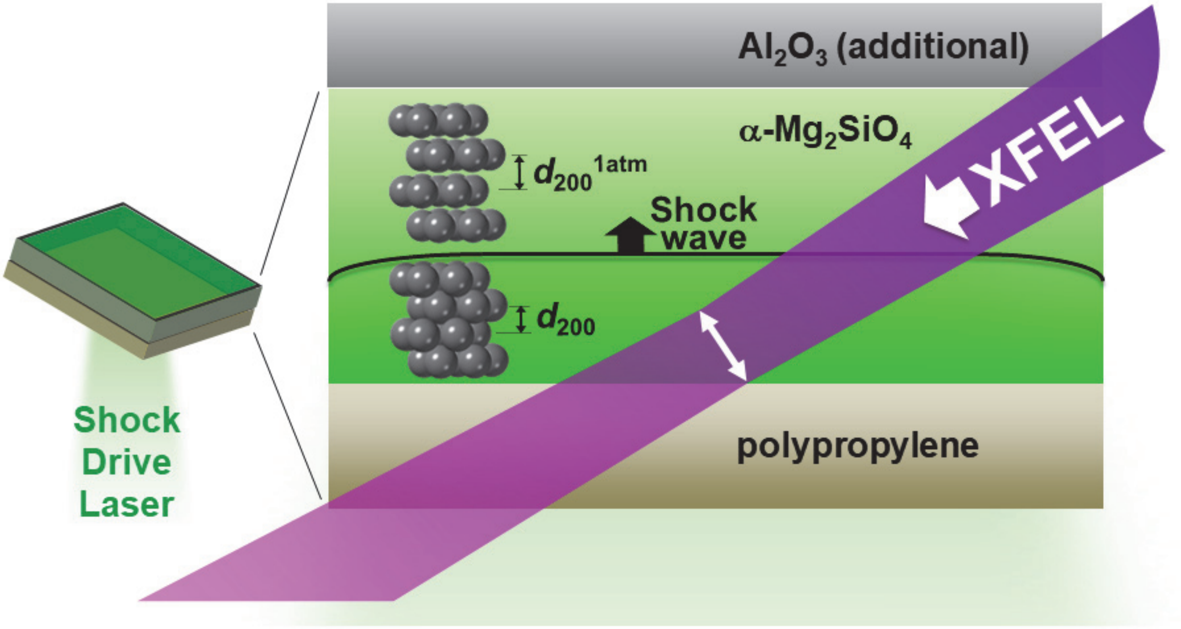

Supplementary Figure 1. Geometry of the XFEL beam passing through the compressed target. The beam of ~15 μm width at its waist (white arrow) was vertically focused to locate its centre at slightly (5 μm) upper position than the interface of ablator / α-Mg_2SiO_4, and was horizontally focused to locate its centre at the centre of the shock drive laser footprint. Therefore, the XFEL beam did not equally probe all the compressed volume, but was rather set to keep probing the most intensely compressed regime of α-Mg_2SiO_4 and its surrounding area when the shock was first injected into the sample. We note that, because of the rarefaction effect coupled with the off-centered XFEL beam trajectory at the interface of α-Mg_2SiO_4 / Al_2O_3, the beam probed extensively-decayed shock in Al_2O_3. We also note that, if the shock drive laser beam was not uniform in space to produce a localized strongly-shocked area, the observed maximum pressure would be much higher than the simulated pressure that is given in Supplementary Fig. 2.

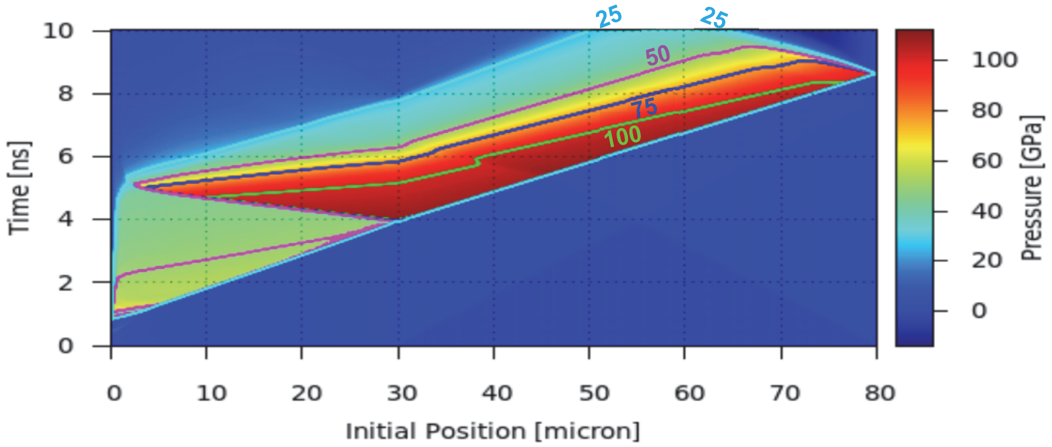

Supplementary Figure 2. A typical result of hydrodynamics simulation conducted using MULTI code[1]. The simulated target was consisted of polypropylene ablator of 30 μm thickness and Mg_2SiO_4 of 50 μm thickness. The laser power density was set at 1.5×10^{12} W/cm^2 for this result. The color lines show pressure contours within the position and time window: green for 100 GPa, blue for 75 GPa, purple for 50 GPa, and light blue for 25 GPa. As shown by these contours, at each measured time by the XFEL beam pulse, there was a large spread of pressure as a function of position, which was detected simultaneously.

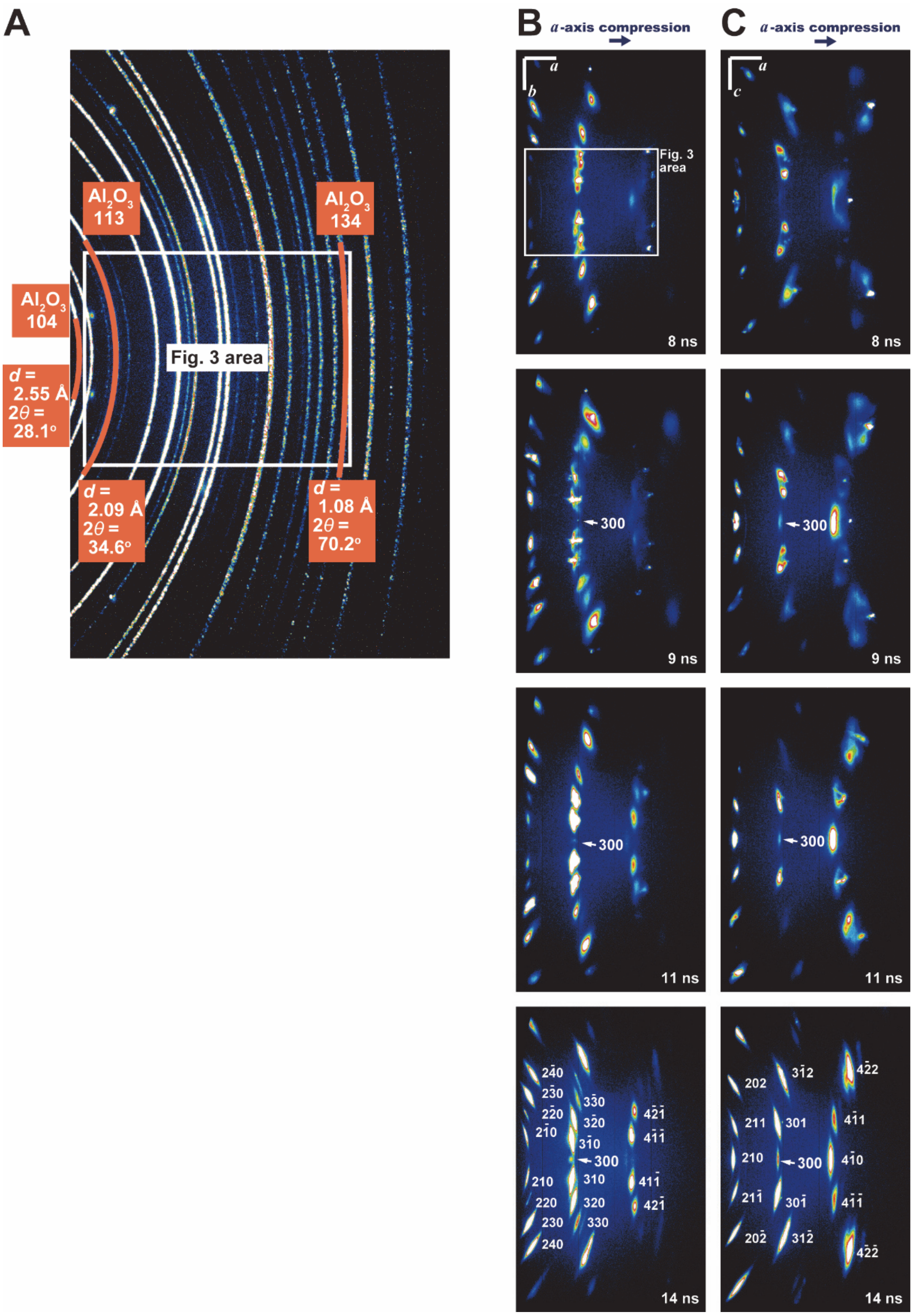

Supplementary Figure 3. The 2θ coverage of the images in Fig. 3 and time evolution of the other reflections around *g* = 300 in the wider-area images. A. The 2θ coverage as calibrated using the polypropylene-olivine-Al_2O_3 layered target before the compression. **B.** The wider-area images taken along the *b* direction. **C.** The wider-area images taken along the *c* direction. Numbers are the Laue indices of Bragg reflections from respective planes of single crystal olivine.

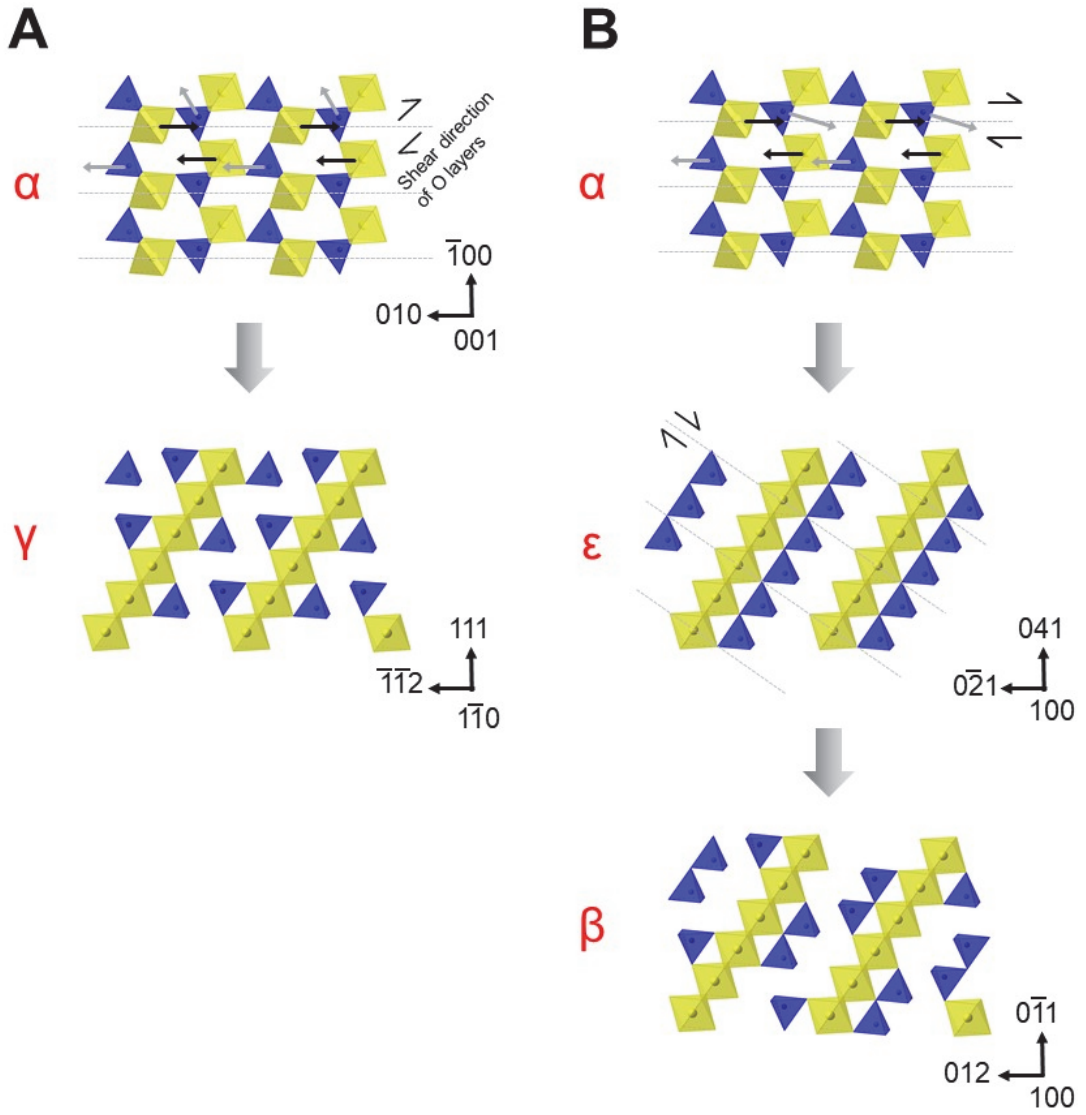

Supplementary Figure 4. Topologies of shear transformation mechanisms of Mg_2SiO_4[2,3]. Polyhedra in yellow and blue are MgO_6 octahedra and SiO_4 tetrahedra, respectively. As described in Figure 4, The α structure is composed of hcp oxygen anions along with their interstitial cations, while the β, γ and ε structures are composed of ccp oxygen anions along with their interstitial cations. The slip planes are indicated by grey broken lines. **A.** The γ structure recrystallizes by slipping of the close-packed oxygen layers on $(100)_\alpha$ slip planes along the *c*-axis direction with $\mathbf{b_p}$ = $1/12[013]_\alpha$, which is one of the dissociated perfect dislocation with $\mathbf{b_t}$ = $[001]_\alpha$. The $\mathbf{b_t}$ is dissociated into two partial dislocations by the following reaction: $[001]_\alpha \rightarrow 1/12[013]_\alpha + 1/12[0{-}19]_\alpha$. The accompanying slight displacements of Mg and Si cations are indicated by the black and grey arrows, respectively. The total shearing process involving these cation's displacements induces disordered spacings of the $(001)_\alpha$ plane which contribute to diffused diffraction spots along the direction normal to that plane (Fig. 3).

B. The ε structure recrystallizes by slipping of the close-packed oxygen layers on $(100)_{\alpha}$ slip planes along the *b*-axis direction with $\mathbf{b_p} = 1/3[010]_{\alpha}$, which is one of the dissociated perfect dislocation with $\mathbf{b_t} = [010]_{\alpha}$..The $\mathbf{b_t}$ is dissociated into two partial dislocations by the following reaction: $[010]_{\alpha} \rightarrow 1/3[010]_{\alpha} + 2/3[010]_{\alpha}$. The accompanying slight displacements of Mg and Si cations are indicated by the black and grey arrows, respectively. The total shearing process involving these cation's displacements induces disordered spacings of the $(010)_{\alpha}$ plane which contribute to diffused diffraction spots along the direction normal to that plane (not observed by the experiments). Further topological consideration suggests that the wadsleyite structure can be formed by subsequent shearing on the $(010)_{\varepsilon}$ plane of the ε structure[2]. The Burgers vector **b** of a dislocation is a crystal lattice vector that quantifies the difference between the distorted lattice around the dislocation (line defect) and the perfect lattice. It denotes the direction and magnitude of the atomic displacement that occurs when a dislocation moves[4].

Supplementary References

1. Ramis, R., Schmalz, R., Meyer-Ter-Vehn, J. MULTI—A computer code for one-dimensional multigroup radiation hydrodynamics. *Comput. Phys. Commun.* **49**, 475–505 (1988).
2. Madon, M. & Poirier, J. P. Transmission electron microscope observation of alpha, beta and gamma $(Mg, Fe)_2SiO_4$ in shocked meteorites: planar defects and polymorphic transitions. *Phys. Earth Planet. Inter.* **33**, 31-44 (1983).
3. Poirier, J. P. Martensitic olivine-spinel transformation and plasticity of the mantle transition zone in *Anelasticity in the Earth* (eds. Stacey, F. D., Paterson, M. S. & Nicholas, A) 113-117 (American Geophysical Union, Washington DC, 1981).
4. Hull, D. & Bacon D. J. *Introduction to Dislocations* (Elsevier, ed. 5, 2011).
